# Exploring the contributions of bed nets, cattle, insecticides and excitorepellency to malaria control: a deterministic model of mosquito host-seeking behaviour and mortality

**DOI:** 10.1016/j.trstmh.2007.04.022

**Published:** 2007-09

**Authors:** Gerry F. Killeen, Thomas A. Smith

**Affiliations:** a Ifakara Health Research and Development Centre, Box 53, Ifakara, Kilombero, Morogoro, United Republic of Tanzania; b Department of Public Health and Epidemiology, Swiss Tropical Institute, Socinstrasse 57, Basel, CH 4002, Switzerland; c School of Biological and Biomedical Sciences, Durham University, South Road, Durham DH1 3LE, UK

**Keywords:** Malaria, Mosquitoes, *Anopheles*, Transmission, Ecology, Prevention, Host–parasite relationship

## Abstract

Domestic and personal protection measures against malaria exposure either divert host-seeking vectors to other hosts or kill those attempting to feed. Here, we explicitly model mosquito host-seeking processes in the context of local host availability and elucidate the impacts and mechanisms of pyrethroid-treated bed nets in Africa. It has been suggested that excitorepellent insecticides could increase exposure of unprotected humans by concentrating mosquito biting activity on this vulnerable group. This worst-case scenario is confirmed as a possibility where vector populations lack alternative hosts, but an approximate ‘break-even’ scenario, with users experiencing little overall change in exposure, is more likely because of increased mosquito mortality while foraging for resources. Insecticidal nets are predicted to have epidemiologically significant impacts on transmission experienced by users and non-users at levels of coverage that can be achieved by sustainable net distribution systems, regardless of excitorepellency or the ecological setting. The results are consistent with the outcome of several randomised controlled trials, predicting enormous reductions in transmission at individual and community levels. As financial support, technology and distribution systems for insecticide-treated nets improve, massive reductions in malaria transmission could be realised.

## 1 Introduction

Insecticidal measures for protection against adult mosquitoes, including treated nets and indoor residual spraying, are amongst the best established and most effective methods for the prevention of malaria (Rozendaal, 1997). The impacts of pyrethroid-treated nets (Lengeler, 2004a, Lengeler, 2004b) and indoor residual spraying (Kouznetsov, 1977, Mabaso et al., 2004) are clearly proven and they remain the most commonly advocated means for individuals and communities to tackle their local malaria problems (Rozendaal, 1997). The recent successes of insecticide-treated nets (ITN) have revitalised interest in vector control as a viable means to reduce malaria burden, even in parts of sub-Saharan Africa where high transmission levels result in extremely stable prevalence, incidence and clinical burden (Smith et al., 2001, Snow et al., 2005).

ITNs protect individuals either by diverting host-seeking vectors to search for a blood meal elsewhere or by killing those that attempt to feed on that person (Fanello et al., 2003, Lindsay et al., 1989, Lindsay et al., 1991, Lindsay et al., 1992, Miller et al., 1991, Pleass et al., 1993). This means that treated nets not only prevent malaria in a protected individual but can also reduce malaria risk in unprotected individuals by suppressing the density (Carnevale et al., 1988, Magesa et al., 1991, Robert and Carnevale, 1991), survival (Carnevale et al., 1988, Magesa et al., 1991, Robert and Carnevale, 1991), human blood indices (Bøgh et al., 1998, Charlwood et al., 2001) and feeding frequency (Charlwood et al., 2001) of vector populations. Conversely, it has been suggested that the excitorepellency of nets could increase the exposure of unprotected humans by concentrating the attentions of host-seeking mosquitoes upon this vulnerable portion of the population (Genton et al., 1994, Lindsay et al., 1992, Lines et al., 1987). Field studies suggest that any such inequitable effects are outweighed by beneficial impacts in whole communities (Binka et al., 1998, Gimnig et al., 2003a, Gimnig et al., 2003b, Hawley et al., 2003, Hewitt et al., 1997, Hii et al., 2001, Howard et al., 2000, Maxwell et al., 2002). Nevertheless, it is theoretically possible that interventions that divert rather than kill mosquitoes could even increase the stability of malaria transmission by increasing vectorial capacity in the most intense foci of transmission (Dye and Hasibeder, 1986, Hasibeder and Dye, 1988, Woolhouse et al., 1997).

Here we extend previously reported kinetic models of mosquito foraging for resources (Killeen et al., 2001, Killeen et al., 2004) by explicitly modelling the processes of host seeking, encounter and attack so that the effects of bed nets and other forms of domestic protection can be explored in detail. Specifically, we investigate the likely impacts of pyrethroid-treated bed nets under conditions where holoendemic malaria is maintained by either *Anopheles gambiae* Giles or *A. arabiensis* Patton in the presence and absence of cattle as alternative hosts.

## 2 Methods

### 2.1 Model framework and design strategy

**Figure 1:**
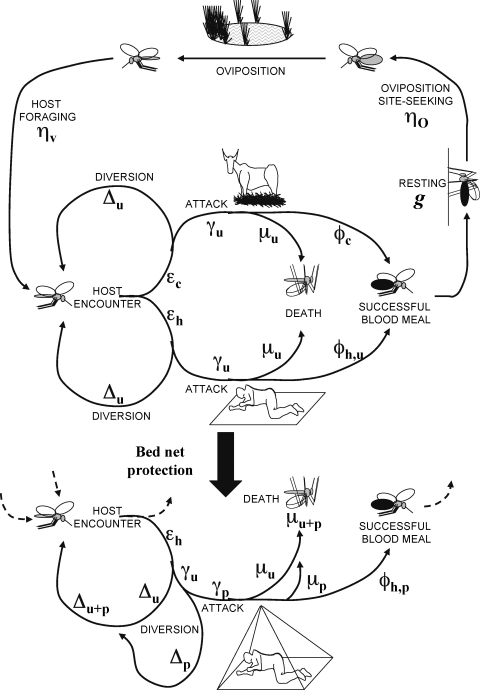
Diagrammatic representation of the model for the mosquito feeding cycle, outlining the rate at which mosquitoes encounter hosts (ɛ) and the probabilities that they will attack (γ), be diverted from (Δ), successfully feed upon (φ) or die (μ) while attempting to feed upon cattle (c) or unprotected humans (h,u). Also depicted are the changes to these probabilities for humans brought about by personal protection with bed nets (p), resulting in overall probabilities of these events for vectors encountering protected humans (h,p).

Although these principles can be extended to allow modelling of transmission by vectors with broader host ranges, here we model the feeding behaviour of the two most common malaria vectors from sub-Saharan Africa. This allows convenient simplification because these species generally feed upon only two host species: *A. gambiae* Giles and *A. arabiensis* Patton feed overwhelmingly upon either humans or cattle (Gillies and Coetzee, 1987, Gillies and DeMeillon, 1968, White, 1974). The latter feed readily upon both humans and cattle, whereas the former greatly prefer humans, particularly in East Africa (Killeen et al., 2001). Here we extend and apply recently developed deterministic models of mosquito host-choice and malaria transmission processes (Killeen et al., 2000a, Killeen et al., 2000b, Killeen et al., 2001, Killeen et al., 2004) to examine the influence of vector behavioural traits and the availability of hosts upon malaria transmission intensity and the success of control measures that target adult mosquitoes. In common with almost all previous models, we assume single populations of humans, cattle and each mosquito species that interact randomly and homogeneously with no gonotrophic discordance (more than one blood meal per gonotrophic cycle (Beier, 1996)). We also assume, as demonstrated for these anthropophagic nocturnal African vector species, that host feeding success is density independent (Charlwood et al., 1995a). The conceptual basis of the model for mosquito behaviour and the effects of bed nets are outlined in Figure 1, and all symbols are defined in Table 1 for clarity and ease of reference. This model adopts a similar conditional probability approach to that previously applied to peridomestic impacts of residual insecticides (Roberts et al., 2000) and is intended to allow easier conceptualisation and parameterisation. The integration of simplified conditional probability models with kinetic (Killeen et al., 2001, Killeen et al., 2004, Saul, 2003) and biodemographic (Carey, 2001, Killeen et al., 2000a, Killeen et al., 2000b, Smith and McKenzie, 2004) components allows further generalisation to consider interactions with the availability of non-human hosts, diversion to unprotected humans, impacts on foraging-associated mortality and estimation of individual- and community-level impacts on human malaria exposure. New formulations, published for the first time in the paper, are outlined in the text of the Methods section, whereas reformulations of published model components are presented for reference in Appendix A.

**Table 1:** Behavioural and host availability input parameters for both vector species

Parameter	Unit	*Anopheles arabiensis*		*Anopheles gambiae*	
		Value	Source	Value	Source
λ_c_	None	1.6	Killeen et al., 2001, White et al., 1972	0.021	Killeen et al., 2001, White et al., 1972
γ_u_	Attacks per encounter with unprotected human or cow^a^^,^^b^	0.90	Lines et al., 1987	0.90	Assumed from Lines et al. (1987)
Δ_u_	Attacks diverted per encounter with unprotected human or cow^a^^,^^b^	0.10	Eq. (2) and Lines et al., 1987	0.10	Eq. (2) and assumed from Lines et al. (1987)
Δ_p_	Additional diversions per protected human attacked^b^	0.35	Lines et al., 1987	0.35	Lines et al., 1987
μ_u_	Mosquito deaths per attack on unprotected humans or cows^a^^,^^b^	0.10	Lines et al., 1987	0.10	Lines et al., 1987
μ_p_	Additional mosquito deaths per protected human attacked^b^	0.50	Lines et al., 1987	0.50	Lines et al., 1987
*a*_h,u_	Successful feeds per day per host-seeking vector per unprotected human^b^	1.2 × 10^−3^	Killeen et al., 2004	1.2 × 10^−3^	Killeen et al., 2004
*a*_c_	Successful feeds per day per host-seeking vector per cow	1.5 × 10^−3^	Killeen et al., 2004	2.5 × 10^−5^	= λ_c_*a*_h,u_
ɛ_h_	Encounters with humans per host-seeking vector per night	1.48 × 10^−3^	Eqs (1) and (3)	1.48 × 10^−3^	Eqs (1) and (3)
ɛ_c_	Encounters with cows per host-seeking vector per night	2.35 × 10^−3^	Eqs (1) and (3)	3.09 × 10^−5^	Eqs (1) and (3)
*E*	Mosquitoes emerging per year	9 × 10^6^	Killeen et al., 2004	9 × 10^6^	Killeen et al., 2004
*κ*	Infected mosquitoes per human bite	0.030	Killeen et al., 2006a	0.030	Killeen et al., 2006a
*N*_c_	Number of cattle	0 or 1000	Killeen et al., 2001	0 or 1000	Killeen et al., 2001
*N*_h_	Number of humans	1000	Charlwood et al., 1995a	1000	Charlwood et al., 1995a
*P*	Proportion surviving per day while resting	0.9	Killeen et al., 2000b, Gillies, 1954	0.9	Killeen et al., 2000b, Gillies, 1954
*P*_ov_	Proportion surviving per day while foraging	0.7–0.9	Saul, 2003	0.7–0.9	Saul, 2003
π_i_	Proportion of normal human exposure during which a net is in use^b^	0.90	Killeen et al., 2006b	0.90	Killeen et al., 2006b

Detailed explanation and justification for all values and definitions are presented in the Methods section.

a Assumed to be identical for both mammalian blood sources.

b Assumed identical for both vector species.

### 2.2 Model description

As defined previously (Killeen et al., 2001), the availability (*a*) of any host (*j*) of any species (*s*) is the product of the rate at which individual vectors encounter it (ɛ_*s*,*j*_) and the probability that, once encountered, they will feed upon it (φ_*s*,*j*_):

(1) $$a_{s,j}=\varepsilon_{s,j}\varphi_{s,j}$$

We now consider successful feeding as just one of three possible outcomes of a host encounter by a female vector, the other two being death while attempting to feed and diversion to seek another host (Figure 1). We consider this as a two-stage process in which the vector first either attacks the encountered host or is diverted away and searches for another, the probabilities of which we denote as γ_*s*_ and Δ_*s*_, respectively. This definition of diversion includes the combined effects of non-contact repellency and contact-mediated irritancy, often referred to as excitorepellency (Muirhead-Thomson, 1960, Roberts et al., 2000). Considering mean values for hosts of any given species, the sum of these two probabilities is:

(2) $$\gamma_{s}+\Delta_{s}=1$$

We then consider that in the second stage of the blood acquisition process, namely feeding, the vector will either feed successfully or die in the attempt, the probabilities of which are denoted by φ_*s*_ and μ_*s*_, respectively. Note that μ_*s*_ therefore denotes the probability of death per attack rather than per encounter and this should be carefully considered when parameterising this model with field data from experimental hut trials (see Section 2.4). Thus, the probability of a successful feed per encounter is the product of two probabilities defined by losses to diversion and death: the successful feeding probability for a vector on any encounter with a host of species *s* is the product of the probability that it will attack that host and the probability that it will survive that attack:

(3) $$\varphi_{s}=\gamma_{s}(1-\mu_{s})=(1-\Delta_{s})(1-\mu_{s})$$

Personal protection measures such as bed nets, repellents or domestic insecticide use will modify these probabilities. We model these effects as a function of the probability that the vector would otherwise successfully feed upon a host of species *s* (Figure 1). The effect of such a protective intervention can also be envisaged as three possible outcomes, the probabilities of which sum to 1: for a vector that would normally choose to feed upon an encountered unprotected human with a probability of φ_h,u_, the presence of a net or other intervention is expected to change this probability to φ_h,p_ as a function of the additional probability of diverting (Δ_p_) and killing (μ_p_) that vector (Figure 1). The overall diversion (Δ_u+p_) and mortality (μ_u+p_) probabilities of protected hosts also depend on whether the host is actually using the net at the time, so the effect of excitorepellency and mortality is adjusted to reflect the proportion of normal exposure during which the host is actually covered (π_i_):

(4) $$\Delta_{\text{u}+\text{p}}=\Delta_{\text{u}}+\Delta_{\text{p}}(1-\Delta_{\text{u}})\pi_{\text{i}}$$

(5) $$\mu_{\text{u}+\text{p}}=\mu_{\text{u}}+\mu_{\text{p}}(1-\mu_{\text{u}})\pi_{\text{i}}$$

Note that the terms Δ_u_ and μ_u_ are included because detailed examination of experimental hut trial results (Lines et al., 1987) indicates that a certain low but clear level of diversion does occur even in the absence of nets. This may be particularly important for future applications focusing on environmental management interventions that limit the availability of resources and are enhanced by increasingly lengthy foraging intervals of mosquitoes (Gu et al., 2006, Killeen et al., 2004).

For any given number of cattle (*N*_c_), unprotected humans (*N*_h,u_) and protected humans (*N*_h,p_), the mean seeking interval for vertebrate hosts (η_v_) can be calculated as the reciprocal of total host availability (*A*) (Killeen et al., 2001), using estimates of these feeding probabilities, their corresponding encounter rates and the corresponding number of hosts of that species (s) or category (*A*_*s*_ = *N*_*s*_ *a*_*s*_) by adapting Eq. (4) of our original formulation (Killeen et al., 2001, Killeen et al., 2004):

(6) $$\eta_{\text{v}}=\frac{1}{A}=\frac{1}{A_{\text{h},\text{u}}+A_{\text{h},\text{p}}+A_{\text{c}}}=\frac{1}{N_{\text{h},\text{u}}a_{\text{h},\text{u}}+N_{\text{h},\text{p}}a_{\text{h},\text{p}}+N_{\text{c}}a_{\text{c}}}$$

where *A*_*s*_ refers to the total availability of all hosts of species *s*. In this case, the species or species categories considered are unprotected humans (h,u), protected humans (h,p) and cattle (c). Values for *a*_c_ and *a*_h,u_ (previously *a*_h_) are estimated exactly as described previously (Killeen et al., 2004; see Table 1 and Section 2.3) and *a*_h,p_ is calculated as follows:

(7) $$a_{\text{h},\text{p}}=\lambda_{\text{p}}a_{\text{h},\text{u}}$$

where λ_p_ is the relative availability of protected versus unprotected hosts, estimated in terms of the ratio of their feeding probabilities:

(8) $$\lambda_{\text{p}}=\frac{\varphi_{\text{h},\text{p}}}{\varphi_{\text{h},\text{u}}}=\frac{\left(1-\Delta_{\text{u}+\text{p}})(1-\mu_{\text{u}+\text{p}}\right)}{\left(1-\Delta_{\text{u}})(1-\mu_{\text{u}}\right)}$$

We adapt Eq. (3) from our previous formulation (Killeen et al., 2004) to estimate the survival rate per feeding cycle (*P*_*f*_) as the product of the probabilities of surviving the gestation (*g*), oviposition site-seeking (η_o_) and vertebrate host-seeking (η_v_) intervals, assuming a constant survival rate of *P* for these intervals, as well as the probability of surviving the eventual attack on a host that may be protected (*P*_γ_):

(9) $$P_{f}=P^{g+\eta \text{o}+\eta \text{v}}P_{\gamma }$$

where the mean probability of mosquitoes surviving their chosen host attack (*P*_γ_) is calculated assuming the proportion of all attacks that end in death is the sum of the mortality probabilities for attacking protected and unprotected hosts weighted according to the proportion of all encounters that will occur on such hosts. Assuming that protection does not affect encounter rates, and that these are proportional to availability when unprotected, we apply this weighting approach to estimate the total attack-related mortality rate and consequent survival as follows:

(10) $$P_{\gamma }=1-\frac{\mu_{\text{u}+\text{p}}a_{\text{h},\text{u}}N_{\text{h},\text{p}}+\mu_{\text{u}}(a_{\text{c}}N_{\text{c}}+a_{\text{h},\text{u}}N_{\text{h},\text{u}})}{a_{\text{h},\text{u}}(N_{\text{h},\text{u}}+N_{\text{h},\text{p}})+a_{\text{c}}N_{\text{c}}}$$

Similarly, the human blood index is calculated as the proportion of total host availability accounted for by humans (Killeen et al., 2001), similarly to Eq. (6):

(11) $$Q_{\text{h}}=\frac{A_{\text{h},\text{u}}+A_{\text{h},\text{p}}}{A_{\text{h},\text{u}}+A_{\text{h},\text{p}}+A_{\text{c}}}$$

The entomological inoculation rate (EIR) for protected and unprotected individuals can then be calculated from the total number of infectious bites upon humans that occur in the population as a whole (*βE*; Killeen et al., 2000a, Killeen et al., 2000b), the share of the total human availability represented by that group and the population size of that group:

(12) $$\text{EI}{\text{R}}_{\text{h},\text{u}}=\frac{\beta E\;A_{\text{h},\text{u}}}{A_{\text{h}}\;N_{\text{h},\text{u}}}$$

(13) $$\text{EI}{\text{R}}_{\text{h},\text{p}}=\frac{\beta E\;A_{\text{h},\text{p}}}{A_{\text{h}}\;N_{\text{h},\text{p}}}$$

where *β* is the mean number of infectious human bites each emerging mosquito takes in its lifetime and *E* is the emergence rate of mosquitoes (Killeen et al., 2000b, Killeen et al., 2004). Dividing Eq. (13) by Eq. (12), substituting with Eq. (7) and rearranging also leads to an intuitively satisfactory solution:

(14) $$\text{EI}{\text{R}}_{\text{h},\text{p}}=\lambda_{\text{p}}\;\text{EI}{\text{R}}_{\text{h},\text{u}}$$

Otherwise, malaria transmission is modelled exactly as described previously (Killeen et al., 2004). This model was adapted from its original formulation (Killeen et al., 2000b) to account for superinfection of mosquitoes (Smith and McKenzie, 2004) and to smooth the effects of changing host availability patterns on feeding cycle length (Killeen et al., 2004). Specifically, the model is adapted to a daily cycle and cumulative survival up to each age (*x*) is estimated as follows and used to calculate the EIR and associated parameters as previously described (Killeen et al., 2000b, Killeen et al., 2004):

(15) $$P_{x}=P_{f}^{x/f}$$

where *f* is the mean feeding cycle length of the vector population. Similarly, the sporozoite infection prevalence of mosquitoes at each age is considered in days, accounting for superinfection:

(16) $$S_{x}=S_{x-1}+\frac{\kappa \;Q(1-S_{x-1})}{f},\text{where }x>n,\text{ otherwise }S_{i}=0$$

where κ denotes the mean infectiousness of the human population to vector mosquitoes (Killeen et al., 2006a) and *n* is the duration of the sporogonic development period of the parasite from ingestion to infective sporozoite stages (Killeen et al., 2000a). Survival and infectiveness probabilities are calculated up to 40 days, after which the contributions of mosquitoes in these age classes to transmission become negligible. Note that *P*_*x*_ is multiplied by *S*_*x*_ to obtain the corresponding probability of being both alive and infective (*I*_*x*_) on each day, and the relevant mosquito lifetime biodemographic parameters required to predict the EIR are calculated by summing these three age-specific outcomes as previously described (Killeen et al., 2000b, Killeen et al., 2004).

### 2.3 Baseline mosquito behaviour, host availability and survival parameters

As an example, we take Namawala in the Kilombero Valley, Tanzania, as a primary centre for parameterising our model because of the exceptionally detailed quantitative characterisation of malaria transmission and vector biodemography in this village and the surrounding area. This is a holoendemic village with intense seasonal transmission, stable high parasite prevalence in humans and a heavy burden of clinical malaria (Charlwood et al., 1995a, Charlwood et al., 1995b, Charlwood et al., 1997, Charlwood et al., 1998, Kitua et al., 1996, Smith et al., 1993, Smith et al., 1995, Smith et al., 1998). This is a site where the bulk of transmission is mediated by *A. gambiae* s.l. (of which the main species involved in transmission is *A. arabiensis*) and where transmission intensity has been modelled with available field data (Killeen et al., 2000a, Killeen et al., 2000b).

As previously (Killeen et al., 2000a, Killeen et al., 2000b), we base our estimate of human population size (Charlwood et al., 1995a) approximately upon those reported for this particular village during the early 1990s. Nevertheless, we use a human population size of 1000 and, where relevant, a bovine population of the same size (Killeen et al., 2001) so that the EIR experienced by users and non-users can be easily calculated at net coverage levels approaching 0% and 100%. By setting coverage to 0.001 or 0.999, this simulates a single user or non-user in the population, respectively. Infectiousness of humans (κ) is set to 0.030, reflecting a more precise recent estimate (Killeen et al., 2006a) than was available previously (Charlwood et al., 1995b, Charlwood et al., 1997). The emergence rate remains set at 9 × 10^6^ emerging vectors per year, as previously described (Killeen et al., 2004). We set mean daily survival of hazards other than feeding (*P*) at 0.90, reflecting a median value of daily survival at four well characterised holoendemic sites (Killeen et al., 2000b) and estimated daily indoor survival for *A. gambiae* s.l. in Tanzania (Gillies, 1954). The results of experimental hut studies (Lines et al., 1987) are combined with host-choice evaluations (White et al., 1972) and appropriate analytical models (Killeen et al., 2001, Killeen et al., 2004) to define the attack and mortality probabilities of *A. arabiensis* encountering cattle or humans: we set the probability that *A. arabiensis* will attack unprotected cattle or humans (γ_u_), conditional upon encountering them, to be 0.90 and the chance that they will die in the attempt (μ_u_) at 0.10. Using these parameters and Eq. (3), we calculate that for *A. arabiensis* the overall feeding probability upon either cattle (φ_c_) or unprotected humans (φ_h_) would be 0.81, a value similar to previous estimates of approximately 0.80–0.85 for the feeding success of *A. gambiae* s.l. upon sleeping humans in Tanzania (Charlwood et al., 1995a, Lines et al., 1987). We also apply these same probabilities of attacking (γ_u_), feeding (φ_h,u_) and dying (μ_u_) to *A. gambiae* s.s. encountering unprotected humans. The availabilities of unprotected humans and cattle are calculated for *A. arabiensis* using field measurements of the duration of the feeding cycle and extended to *A. gambiae*, accounting for the lower estimated relative availability of cattle (λ_c_) to this mosquito species as previously described (Killeen et al., 2001; Table 1). Note that λ_c_ is assumed to modify *a*_c_ by affecting the encounter rate only, indicating that these mosquitoes can differentiate between preferred and non-preferred hosts at long range (Gillies and Wilkes, 1969, Gillies and Wilkes, 1970, Gillies and Wilkes, 1972). In the case of *A. arabiensis*, this assumption is consistent with the longer range of attraction of cows relative to humans for zoophilic members of the *A. gambiae* complex (Gillies and Wilkes, 1969, Gillies and Wilkes, 1970, Gillies and Wilkes, 1972).

### 2.4 Parameters reflecting the effects of insecticide-treated bed nets

The effects of ITNs upon feeding probability and mortality risk of either *A. gambiae* s.l. sibling species encountering a protected human are assumed to be identical and are derived from the results of detailed experimental hut trials from northern Tanzania (Lines et al., 1987). Trials with *A. arabiensis* were carried out in Magugu, west of Arusha, under experimental conditions that excluded cattle from the immediate surroundings, and with *A. gambiae* in Muheza, near Tanga. In this study, nylon nets were impregnated with 0.2 g/m^2^ permethrin and their effects on house entry, feeding success and survival were quantified. Combining results from nets with and without holes, these authors reported that, of vectors that would otherwise feed successfully, the proportion that successfully fed and survived the hazards of the treated net (φ_p_) was approximately 0.11–0.17. This reduced feeding and survival success was attributed to an increased mortality (μ_p_) of approximately 0.50–0.55, implying that the proportion of vectors being diverted by the nets (Δ_p_) was approximately 0.27–0.38. Based on these estimates, we set μ_p_ and Δ_p_ at 0.50 and 0.35, respectively (Table 1). Although the ability of permethrin to divert or kill vectors varies considerably with formulation (Lindsay et al., 1991, Pleass et al., 1993), the values we have chosen compare well with those from other studies applying similar permethrin doses in East and West Africa (Mathenge et al., 2001, Pleass et al., 1993). The proportion of normal biting exposure that occurs while nets are actually in use (π_i_) has been estimated as 90% for *A. gambiae* in southern Tanzania (Killeen et al., 2006b), so we set π_i_ to a value of 0.90. Note that unlike previously published applications of this model (Killeen et al., 2006b), here we use π_i_ rather than π_s_, the proportion of bites that occur during peak sleeping hours, because the former more comprehensively captures the level of protection afforded by a net.

### 2.5 Testing the sensitivity of the conclusions to increased mosquito mortality while foraging for resources

Foraging for resources is an intrinsically dangerous undertaking for mosquitoes and it is almost certain that survival during these phases is lower than while resting in houses (Kelly and Thompson, 2000, Saul, 2003). Assuming this is the case, the simulations we have outlined thus far will underestimate the impact of diversionary measures for non-users because these interventions are expected to have a larger impact on mosquito survival, sporozoite prevalence and EIR if foraging is more hazardous than resting. We therefore tested the sensitivity of our conclusions to deviations from the assumption that mortality is constant across all phases of the life cycle. This was accomplished by separately considering survival of the resting (*P*), foraging (*P*_ov_) and attacking (*P*_γ_) phases and adapting Eq. (9) accordingly:

(17) $$P_{f}=P^{g}P_{\text{ov}}^{\eta \text{o}+\eta \text{v}}P_{\gamma }$$

where *P*_ov_ is an assumed common survival rate for mosquitoes foraging for either oviposition sites or vertebrate hosts. Values for *P*_ov_ were varied from 0.9 (equivalent to the formulation described above in Eq. (9)) down to 0.7, reflecting a potentially realistic range of foraging survival rates under field conditions (Saul, 2003). This range of *P*_ov_ was evaluated in terms of its impact on the protection afforded to unprotected individuals in populations where 75% of the human population used nets that diverted, killed, or diverted and killed mosquitoes. This analysis was conducted for both of the two distinct scenarios described above: *A. gambiae* s.l. in the absence of alternative hosts and *A. arabiensis* in the presence of cattle.

## 3 Results

**Figure 2:**
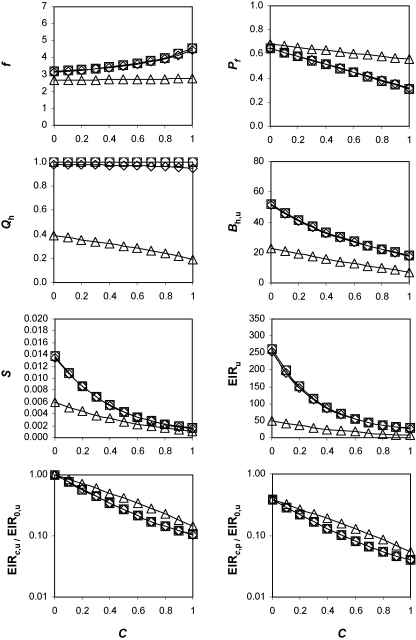
Predicted effects upon malaria transmission intensity of insecticide-treated nets that both divert and kill mosquitoes. The diversionary and insecticidal properties of the nets are as described in experimental hut trials (Lines et al., 1987) and are summarised in Section 2.4. The outcome variables plotted on the *y*-axes are the survival probability per feeding cycle (*P*_*f*_; Eq. (9)), the human blood index (*Q*_h_; Eq. (11); human bites per bite), the feeding cycle length (*f*; Eq. (A.1); nights), the biting rate experienced by unprotected humans (*B*_h,u_; Eq. (A.5); bites per person per night), the sporozoite prevalence (*S*; Eq. (A.4); infectious bites per bite), the entomological inoculation rate of an unprotected human (EIR_u_; Eq. (12); infectious bites per person per year) and the relative exposure of unprotected community members (EIR_*c*,u_/EIR_0,u_; Eq. (12)) as well as protected members using nets (EIR_*c*,p_/EIR_0,u_; Eqs (12) and (13)). These outcomes are plotted as a function of increasing levels of coverage with effectively treated nets (*C*, expressed in terms of net use) for *Anopheles gambiae* and *A. arabiensis* vector populations in the presence and absence of one head of cattle per person: *A. arabiensis* without cattle (□), *A. arabiensis* with cattle (▵), *A. gambiae* s.s*.* without cattle (○) and *A. gambiae* s.s*.* with cattle (◇).

First we compare the predicted effects of increasing coverage of effectively-treated nets with both diversionary and insecticidal properties in four different scenarios: pure populations of either *A. arabiensis* or *A. gambiae* s.s. in the presence or absence of cattle (Figure 2).

The individual protection afforded to users is constant for all scenarios and coverage levels because this component of protection only occurs in and around the domestic environment where the parameter values, as well as the entities and processes they describe, are assumed identical. Nets are predicted to prevent 57% of exposure amongst users. This is somewhat lower than previous field estimates of 69% at a northern Tanzanian site very close to some of those used to parameterise this model (Soremekun et al., 2004). Although this difference might be partially explained by the modest excitorepellent and insecticidal properties we have assumed for treated nets, complementary behavioural studies indicate approximately 10% of normal exposure occurs outdoors (Killeen et al., 2006b). The inability of nets to prevent this directly is unlikely to be captured by the estimates of Soremekun et al. (2004) because only mosquitoes that entered houses were sampled. Thus, our simulation of protection against all exposure appears quite compatible with the estimate of Soremekun et al. (2004), which probably reflects protection against indoor exposure only.

Next, we go beyond the direct protection afforded to individual users to consider community-level protection through impacts on vector–parasite biodemography (Figure 2). The presence of cattle has a substantial influence on the effects of nets only for *A. arabiensis*, so all subsequent comparisons consider only two scenarios: (1) a population of either species (*A. gambiae* s.l.) in the absence of cattle; and (2) an *A. arabiensis* population in the presence of cattle. In all scenarios described in Figure 2, ITNs reduce transmission both for users and non-users by lowering mosquito survival. Nets also act by extending the length of the mosquito feeding cycle in all scenarios, except for *A. arabiensis* in the presence of cattle. In this case, however, the failure to increase feeding cycle length results from diversion of mosquitoes to cattle so that the human blood index, and hence transmission intensity, is reduced. The availability of cattle as an alternative host to *A. arabiensis* does somewhat reduce the impact of nets on mosquito survival and EIR. In all cases, however, epidemiologically significant impacts on EIR of users and, to a lesser extent, non-users are predicted at levels of coverage that have already been achieved through established delivery mechanisms. As proposed based on large-scale field trials (Hawley et al., 2003), absolute coverage of 50% use of effectively-treated nets is expected to achieve useful community-wide protection of non-users in all scenarios, and increasing gains are realised as coverage is increased further. Specifically, the lowest predicted level of protection against exposure was for non-users and *A. arabiensis* in the presence of cattle, but even this 58% reduction at 50% coverage closely approaches the 60–70% reductions thought to account for the personal protection of ITNs against malarial disease, regardless of the local endemicity level (Killeen et al., 2006b, Soremekun et al., 2004). We note that sustained large-scale net distribution systems in the Kilombero Valley, southern Tanzania, have exceeded this target and achieved 75% net use (Killeen et al., unpublished data) using hybrid social marketing systems that deliver targeted public sector subsidies for ITNs that are obtained through the private sector (Magesa et al., 2005, Mushi et al., 2003, Schellenberg et al., 2001).

**Figure 3:**
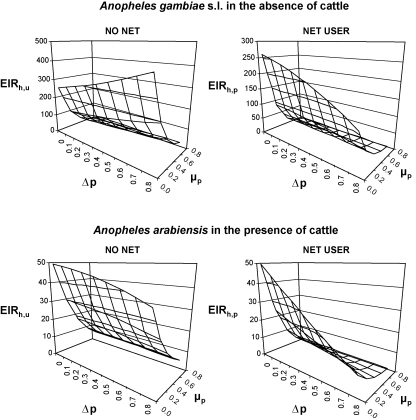
Impacts of insecticide-treated nets on malaria transmission as a function of their ability to divert and kill host-seeking mosquitoes. Malaria transmission intensity (entomological inoculation rate) for individuals with (EIR_h,p_; Eq. (13)) and without (EIR_h,u_; Eq. (12)) nets is plotted as a function of their ability to divert (Δ_p_) and kill (μ_p_) mosquitoes attacking protected humans. The results presented represent simulations assuming 75% usage of nets in two distinctive scenarios: *Anopheles gambiae* s.l. in the absence of cattle (results for both sibling species are identical) and *A. arabiensis* in the presence of one head of cattle per person.

In the second set of scenarios, we explore the impacts of nets with varying levels of diversionary and insecticidal properties upon the EIR (Figure 3) at a coverage rate of 75%, consistent with recent programmatic observations in southern Tanzania (Killeen et al., unpublished data). All formulations confer protection to users and non-users alike for *A. arabiensis* in the presence of cattle. However, the use of excitorepellent formulations in the absence of cattle actually appears to increase the malaria transmission intensity experienced by non-users, regardless of the vector species or insecticidal properties of the net. Thus, the worst-case scenario (Genton et al., 1994, Lindsay et al., 1992, Lines et al., 1987), in which the impacts of excitorepellency on mosquito survival, feeding cycle length and human feeding frequency are outweighed by the concentration of remaining transmission upon the most vulnerable, appears to be possible under these conditions. However, this does not appear to be a realistic scenario given the proven impact of ITNs for non-users regardless of the degree of anthropophily of local vector populations (Binka et al., 1998, Gimnig et al., 2003a, Gimnig et al., 2003b, Hawley et al., 2003, Hewitt et al., 1997, Hii et al., 2001, Howard et al., 2000, Maxwell et al., 2002). Examining the simulated situation more closely, the assumption of constant mosquito mortality throughout the gonotrophic cycle is crucial and almost certainly underestimates the impact of diversion on mosquito longevity and community-level transmission for users and non-users alike (Kelly and Thompson, 2000, Saul, 2003).

**Figure 4:**
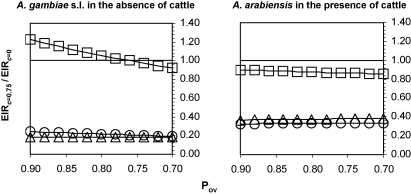
Influence of reduced mosquito survival during foraging (*P*_ov_; Eq. (17)) on the protection of non-users (Eq. (12)) by nets that divert (□), kill (▵) or divert and kill (○) mosquitoes. We assume 75% coverage with nets (*C* = 0.75; Killeen et al., unpublished data) that cause 40% diversion (Δ_p_ = 0.4) and/or mortality (μ_p_ = 0.4) of mosquitoes in two scenarios: *Anopheles gambiae* s.l. in the absence of alternative hosts and *A. arabiensis* in the presence of cattle. Protection is expressed in terms of reduction in the entomological inoculation rate (EIR) relative to conditions without any nets (*C* = 0) in the community at the same value of survival during foraging (*P*_ov_).

We therefore tested the sensitivity of our predictions to increased mosquito mortality during the active foraging phase of the host-seeking interval. Figure 4 reveals just how insensitive the efficacies of all formulations are to more realistic levels of foraging-associated mortality (*P*_ov_). The communal protection afforded to non-users in populations with high coverage of ITNs is completely insensitive to *P*_ov_ for all nets that are insecticidal, regardless of the vector system. Protection of non-users by purely excitorepellent ITNs is minimal and similarly insensitive to *P*_ov_ for human populations exposed to zoophilic vectors such as *A. arabiensis* in the presence of alternative hosts. For human-dependent *A. gambiae* in the absence of alternative hosts, very low values of *P*_ov_ are required to kill diverted mosquitoes fast enough to prevent increased risk among non-users. If the mosquito survival rate while foraging approaches that measured while resting indoors, purely excitorepellent nets could increase the exposure of unprotected humans by concentrating the attentions of host-seeking mosquitoes upon this vulnerable portion of the population (Genton et al., 1994, Lindsay et al., 1992, Lines et al., 1987). Such an eventuality could even increase the stability of malaria transmission by increasing vectorial capacity in the most intense foci of transmission (Dye and Hasibeder, 1986, Hasibeder and Dye, 1988, Woolhouse et al., 1997). Whilst we suggest that such high survival rates for foraging mosquitoes are highly unlikely (Kelly and Thompson, 2000, Saul, 2003), setting lower *P*_ov_ values suggests an approximately break-even outcome for non-users where vector populations lack alternative hosts. Below the median plausible foraging survival rate of 0.8 per day, high coverage with ITNs results in higher biting rates upon non-users but lower sporozoite prevalence so that no substantial change of EIR is expected.

**Figure 5:**
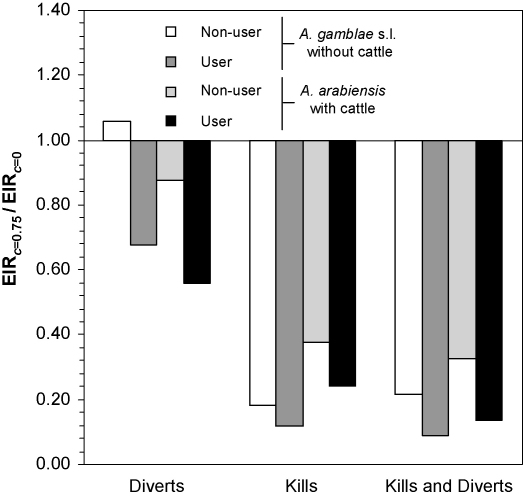
Impact of nets that divert, kill, or divert and kill mosquitoes on transmission intensity experienced by users and non-users (Eqs (12) and (13)). We assume 75% coverage with nets (Killeen et al., unpublished data) that cause 40% diversion (Δ_p_ = 0.4) and/or mortality (μ_p_ = 0.4) in two distinct scenarios: *Anopheles gambiae* s.l. in the absence of alternative hosts and *A. arabiensis* in the presence of cattle. Impact is expressed in terms of reduction in the entomological inoculation rate (EIR) relative to conditions without any nets (*C* = 0) elsewhere in the community. Mosquito survival while foraging (*P*_ov_; Eq. (17)) was set to a median plausible value of 0.80 per day.

Figure 5 illustrates this point more succinctly. All combinations of excitorepellency and insecticidal properties will protect users against exposure, but those lacking insecticidal properties will provide little or no communal protection and will therefore fail to protect non-users. Excitorepellency will always enhance the protection of ITN users and is expected to improve protection of non-users where alternative hosts are available but to slightly attenuate this benefit where humans are the only available host. We nevertheless suggest that excitorepellency will improve the effectiveness of insecticidal ITNs, even in the latter scenario, because this will enhance the personal protection that motivates individual uptake and subsequently increases community-level coverage.

## 4 Discussion

Here we have outlined a kinetic mosquito behaviour and mortality model, parameterised it with field measurements of its component processes, and predicted impacts on malaria transmission that are consistent with the results of large-scale randomised controlled trials. Extending these results to consider vector populations that are resistant to the insecticidal properties of net treatments but not their excitorepellent properties (Potikasikorn et al., 2005) supports the view that treated nets can provide personal protection even when they fail to kill target mosquito species (Fanello et al., 2003). Nevertheless, the model suggests that purely diversionary interventions will probably have minimal impacts on community-level transmission and the exposure of non-users. The worst-case scenario in which high coverage with purely excitorepellent interventions increases exposure of non-users is theoretically unlikely but not impossible. In practice, it has been observed that pyrethroid-treated ITNs can even benefit non-users where *kdr* resistance alleles occur at high frequencies (Henry et al., 2005), although it should be noted that West African *A. gambiae* may be more zoophilic than the East African populations used to parameterise this model (Killeen et al., 2001). We therefore suggest that operational programmes delivering domestic interventions such as ITNs or indoor residual sprays should prioritise products based on their insecticidal properties but also opt for formulations that maximise excitorepellency and personal protection.

This model and argument could just as easily be applied to indoor residual spraying of dichlorodiphenyltrichloroethane (DDT), a comparably efficacious intervention (Curtis and Mnzava, 2000) with similar impacts upon the survival of *A. gambiae* (Magesa et al., 1991). Roberts et al. (2000) consider that DDT functions effectively, even against resistant zoophagic vector populations in Thailand, India and Mexico (Potikasikorn et al., 2005, Roberts and Andre, 1994), because it acts largely through excitorepellent diversion rather than direct killing. Thus, the approach described here may be particularly useful for elucidating the mechanisms and effects of such largely diversionary intervention strategies. Unlike previous models based on experimental hut studies alone (Roberts et al., 2000), the survival cost of extended dispersal periods outside of houses can be simulated and its contribution to the overall impact can be predicted in quantitative terms. This model may also be developed to assess alternative strategies such as insecticide treatment of cattle (Kawaguchi et al., 2004, Rowland et al., 2001, Saul, 2003). Although irrelevant in some situations (Charlwood et al., 1995a), density-dependent feeding success has been documented for these vectors (Lindsay et al., 1995) and this potential complication should be considered before extending to other host–vector systems (Kelly and Thompson, 2000). It should be noted, however, that the kind of excitorepellency we simulate here is a long-lasting property of the net and similar impacts are unlikely to be achieved through the application of volatile substances such as diethyltoluamide (DEET) (Pennetier et al., 2005) because these formulations were found to have only a short persistence when applied to bed nets (Curtis et al., 1987).

Unfortunately, most nets in Africa have, until recently, been non-insecticidal in practice (Roll Back Malaria Partnership, 2005a) and typically in poor condition with many holes under normal conditions of use (Erlanger et al., 2004, Maxwell et al., 2006a). Untreated nets under typical field conditions (Erlanger et al., 2004, Maxwell et al., 2006a) confer little, if any, protection (Curtis et al., 1996, Lines et al., 1987), so national programmes throughout Africa should now focus on achieving high coverage of target groups with nets that remain repellent and insecticidal throughout their lifetime (Roll Back Malaria Partnership, 2005b). This can be achieved by distributing long-lasting insecticidal nets or by applying long-lasting insecticide formulations to existing nets that would otherwise remain ineffective (Asidi et al., 2004, Asidi et al., 2005, Curtis et al., 1996, Graham et al., 2005, Maxwell et al., 2006b, Tami et al., 2004, Yates et al., 2005). Many of the simulations described here have been based on conservative estimates of the insecticidal and diversionary properties of nets, consistent with modest levels of treatment under operational conditions and with treatment technologies as they were two decades ago. Today, long-lasting insecticide treatments with much better insecticidal properties (Asidi et al., 2004, Asidi et al., 2005, Curtis et al., 1996, Graham et al., 2005, Maxwell et al., 2006b, Yates et al., 2005) that are retained for up to 7 years of normal use (Tami et al., 2004) as well as durable net materials that that can last up to 15 years (Killeen, unpublished data) are becoming a proven reality.

Whilst the choice of delivery strategy for ITNs has been contentious in recent years (Curtis et al., 2003, Lines et al., 2003), consensus is emerging that public sector (Anonymous, 2006, Grabowsky et al., 2005) and market-based approaches (Lines et al., 2003, Webster et al., 2005) as well as hybrid systems (Magesa et al., 2005, Mushi et al., 2003) merit investigation, development and long-term evaluation on scales for which no precedent yet exists (Roll Back Malaria Partnership, 2005b). Programmatic-scale evaluations in southern Tanzania suggest hybrid social marketing systems, which combine the strengths of both public sector and market-based systems (Magesa et al., 2005, Mushi et al., 2003, Schellenberg et al., 2001) to achieve high population-level coverage with nets (Killeen et al., unpublished data), could be greatly enhanced by these exciting new ITN technologies. If these same levels of coverage can be achieved with long-lasting insecticidal nets rather than ordinary, poorly maintained and largely untreated nets (Erlanger et al., 2004; Killeen et al., unpublished data; Maxwell et al., 2006a), it may soon be possible to realise the massive malaria transmission reductions predicted under the ideal conditions presented in the right-hand corner of each panel in Figure 3. As global support for malaria control in Africa continues to grow (Haines and Cassels, 2004, Millennium Development Project, 2005) and scale-up of ITN use proceeds across Africa (Roll Back Malaria Partnership, 2005a, Roll Back Malaria Partnership, 2005b), it should be possible to translate theory into practice.

## Authors’ contributions

GFK conceived the model and then developed and applied it with assistance from TAS. GFK drafted the manuscript in consultation with TAS and both authors read and approved the final version. GFK and TAS are guarantors of the paper.

## Funding

Swiss National Science Foundation (award number 2200C0-105994) and the Bill and Melinda Gates Foundation (award number 39777) are thanked for financial support. GFK is supported by the Wellcome Trust through a Research Career Development Fellowship (award number 076806).

## Conflicts of interest

None declared.

## Ethical approval

Not required.

## Appendix A

Here, we briefly outline previously described equations using symbols and terminology revised in accordance with those presented in the Methods section of this paper. The feeding cycle length (*f*) is calculated as the sum of the durations of the gestation period (*g*), the oviposition site-seeking interval (η_o_) and the vertebrate host-seeking interval (η_v_):

(A.1) $$f=g+\eta_{\text{o}}+\eta_{\text{v}}$$

Adapting Eq. (2) from Killeen et al. (2000b) to the nightly time increment described in Eq. (15), the number of human bites the average mosquito takes in a lifetime (*b*_h_) is defined as the sum of the probabilities of surviving and feeding on a human at each age (*x*):

(A.2) $$b_{\text{h}}=Q_{\text{h}}\sum_{x}^{\infty }\frac{P_{\text{f}}^{\text{x}/\text{f}}}{f}$$

Similarly, accounting for superinfection as described in Eq. (16), the number of infectious bites on humans per mosquito lifetime (*β*) is calculated as the product of the human blood index and sum of the products of the probabilities of biting and being infectious at each age (Killeen et al., 2000b):

(A.3) $$\beta =Q_{\text{h}}\sum_{x}^{\infty }S_{\text{x}}\frac{P_{\text{f}}^{\text{x}/\text{f}}}{f}$$

The overall sporozoite prevalence can then be calculated as the quotient of *b*_h_ and *β* (Killeen et al., 2000b; Eq. (14)):

(A.4) $$S=\frac{b_{\text{h}}}{\beta }$$

Replacing *β* with *b*_h_ in Eq. (12), the biting rate experienced by unprotected (*B*_h,u_) and protected humans (*B*_h,p_) is calculated as previously described (Killeen et al., 2000b):

(A.5) $$B_{\text{h},\text{u}}=\frac{b_{\text{h}}\;E\;A_{\text{h},\text{u}}}{A_{\text{h}}\;N_{\text{h},\text{u}}}$$

(A.6) $$B_{\text{h},\text{p}}=\frac{b_{\text{h}}\;E\;A_{\text{h},\text{p}}}{A_{\text{h}}\;N_{\text{h},\text{p}}}$$
